# High-speed running and injury risk in soccer: a systematic review

**DOI:** 10.3389/fpubh.2026.1798241

**Published:** 2026-03-06

**Authors:** Yongli Xie, Xiaofei Cai

**Affiliations:** 1 College of Sport Science, Hefei Normal University, Hefei, Anhui, China; 2 Department of General Education, Anhui Finance & Trade Vocational College, Anhui Economics & Management College, Hefei, Anhui, China

**Keywords:** football, high intensity demands, injury, monitoring, training load

## Abstract

**Background:**

High-speed running (HSR) and sprint exposure are monitored in soccer, but associations with injury are uncertain.

**Objective:**

To synthesis evidence on associations between HSR exposure and injury risk/occurrence, and to explore heterogeneity.

**Methods:**

PubMed, Scopus and Web of Science were searched. No date or language restrictions were applied. Eligibility criteria included observational studies in soccer quantifying training and/or match HSR/sprinting using microtechnology and reporting extractable associations with injury outcomes. Risk of bias was assessed with QUIPS. Narrative synthesis was undertaken without meta-analysis.

**Results:**

From 3,824 records, 22 studies were included. Most were professional/elite and used GPS with absolute speed thresholds. Outcomes mainly involved time-loss or non-contact soft-tissue injuries. Across studies using relative change measures (e.g., acute:chronic contrasts or injury-week vs. control comparisons), short-horizon increases or disproportionate HSR/sprint exposure relative to recent history more often aligned with higher injury risk. In contrast, studies analyzing absolute weekly HSR volume more frequently reported negligible or inconsistent associations within typical exposure ranges, with no reproducible dose threshold emerging. Overall risk of bias was high in 20/22 studies.

**Conclusions:**

Sudden increases in HSR/sprint-related load may be associated with injury, but heterogeneity and bias limit certainty and preclude definitive thresholds.

**Registration:**

OSF (osf.io/wquh2).

## 1 Introduction

Professional soccer is associated with a substantial injury burden, with pooled estimates in men's professional football indicating an overall incidence of approximately 8.1 injuries per 1,000 h of exposure and markedly higher injury incidence during matches than training (1). Lower-extremity injuries predominate and muscle/tendon injuries constitute the most common injury type in professional football players (2). To ensure interpretability and comparability across studies, consensus-based definitions and standardized reporting of injury, severity, and exposure in football injury surveillance have been recommended for nearly two decades (3). Accordingly, we can consider injury definitions (e.g., time-loss vs. medical-attention), severity reporting, and exposure denominators (training and/or match exposure hours where available) against football-specific consensus guidance to support comparability when grouping and interpreting results (3).

Concurrently, the physical demands of elite soccer have evolved toward greater volumes and frequencies of high-intensity running and sprinting during match play across recent seasons (4). High-intensity running profiles vary by position and fluctuate within matches in ways consistent with fatigue and contextual constraints, underscoring the relevance of high-speed locomotor exposure to both performance and health outcomes (5). Within this context, hamstring injuries represent a major component of the time-loss injury burden in men's professional football and have increased over long-term surveillance, reaching approximately one-quarter of all recorded injuries in recent seasons in elite European club football (6). Video and prospective observational evidence in professional football further indicates that many hamstring injuries occur during high-velocity actions and may be preceded by short periods of unusually elevated running demands at high speeds (7, 8).

High-speed running (HSR) exposure in soccer is typically quantified as part of external load monitoring using wearable microtechnology (e.g., GPS/GNSS), for which validity and reliability in team-sport settings have been extensively evaluated (9). Even with modern sampling frequencies, measurement error tends to increase as movement speed rises, which is particularly relevant for high-speed running and sprint metrics that are central to contemporary load-management strategies (10). However, there is no universal consensus regarding the absolute speed thresholds used to define high-speed running and sprinting in adult soccer, leading to substantial between-study variability in reported exposure (11). Although the 19.8 km/h value has been popularized for men, this threshold is population-specific and depends on context. It has been observed across individuals with different training statuses and fitness levels, and it may also differ in other populations such as women and youth (11). Individualized speed zones based on player-specific reference speeds (e.g., maximal sprint speed or fitness-derived thresholds) can meaningfully change quantified high-speed exposures, and such methodological choices may also influence observed associations with injury outcomes (12, 13).

Contemporary conceptual models propose that workload contributes to injury through interacting pathways that include exposure to inciting events, fatigue-related decrements, and fitness-related protective adaptations (14). In line with this framework, rapid increases in training load have been associated with higher likelihood of non-contact soft-tissue injury, whereas well-developed chronic load capacity may be protective (15). In elite soccer specifically, observational cohort evidence has linked large and rapid increases in high-speed running and sprint distances to increased odds of injury, with higher chronic loads and better intermittent aerobic fitness potentially moderating risk (16). Acute:chronic workload ratio approaches have been widely applied in professional soccer, yet football-specific systematic evaluations highlight substantial methodological heterogeneity that complicates causal interpretation and synthesis (17, 18). More generally, prior syntheses across field and team sports conclude that inconsistent injury definitions, workload constructs, and statistical decisions limit confidence in pooled inferences (19). In contrast to these broader workload reviews, the present review focuses specifically on HSR and sprint exposure as the prognostic factor of interest in soccer, explicitly separates evidence on injury risk (modeling/prognostic designs) from injury occurrence (event- or context-focused designs), and structures synthesis around threshold definitions (absolute vs. individualized) and exposure windows (acute vs. chronic) to explain heterogeneity.

Recent professional football studies suggest that both inadequate competitive exposure (including reduced high-speed running exposure) and the strategic provision of near-to-maximal speed running bouts may influence subsequent muscle and hamstring injury risk, implying potentially non-linear dose–response relationships that depend on exposure history and timing (20, 21). Complementary evidence indicating a protective association between higher preseason sprint-related workloads and in-season hamstring injury risk further reinforces the practical importance of clarifying how high-speed running exposure relates to injury outcomes across seasonal phases (22). Nevertheless, fragmentation across speed-threshold definitions, exposure windows (acute vs. chronic), and injury surveillance definitions continues to limit the translation of the literature into robust, generalizable load-management recommendations for soccer (23). These inconsistencies reflect not only divergent findings, but also recurrent methodological limitations, particularly incomplete control for confounding, variability in analytical decisions (e.g., exposure window selection, metric derivation, and model specification), and inconsistent HSR/sprint exposure definitions and processing choices, which reduce comparability and increase risk of biased or non-transportable estimates.

Moreover, the available evidence linking HSR/sprint exposure to injury has historically been dominated by men's elite samples, with comparatively fewer data in women and youth. In senior women's football, injury incidence is also substantial, but the extent to which HSR/sprint-derived external-load constructs relate to injury outcomes remains less well established due to limited study volume and heterogeneity in exposure definitions (24). In youth football, injury incidence varies widely by age and context, and similarly, evidence connecting HSR/sprint exposure to injury is comparatively sparse and methodologically inconsistent (25). Importantly, the literature may classify injury in two analytically distinct ways (26): (i) injury risk, where models estimate the probability/odds of subsequent injury from prior HSR/sprint exposure (prognostic or predictive inference), and (ii) injury occurrence, where analyses are event- or context-based (e.g., contrasting exposure in injury weeks vs. control periods) and are primarily descriptive of circumstances surrounding injury events.

Accordingly, this systematic review aims to synthesize evidence on the associations between players' naturally occurring exposure to high-speed running (training and match; absolute and individualized thresholds; acute and chronic metrics) and both injury risk and injury occurrence in soccer players, and to identify methodological and contextual factors that may explain heterogeneity across studies. To match these differing inferential aims, we synthesis prognostic/predictive injury risk studies separately from event-based/descriptive injury occurrence studies, rather than treating them as estimating the same causal estimand. We prespecified heterogeneity exploration across effect modifiers commonly implicated in load–injury models, including exposure context (training vs. match vs. combined), exposure window (acute vs. chronic vs. acute:chronic contrasts), injury phenotype (all injuries vs. non-contact soft-tissue/muscle injuries vs. hamstring injuries), and competitive level/age group (youth vs. adult; elite/professional vs. sub-elite).

## 2 Methods

This systematic review protocol was developed and reported in accordance with the PRISMA 2020 statement (27), and the planned methods were specified and registered a priori to reduce risk of selective decision-making during screening, extraction, and synthesis. Registration was conducted in the Open Science Framework platform (osf.io/wquh2; date: 20/01/2026).

### 2.1 Eligibility criteria

Studies were eligible if they included soccer players (any sex, age group, and competitive level) and examined players' exposure to high-speed running (HSR) and/or sprinting during routine training and/or match play (i.e., naturally occurring external load exposure), quantified using wearable microtechnology (e.g., GPS/GNSS, local positioning systems) or equivalent time–motion tracking methods, and related these exposures to injury outcomes. Eligible study designs were non-interventional analytical observational studies, including prospective or retrospective cohort studies, case–control studies, and analytic cross-sectional studies, because these designs aligned with the review objective of estimating associations between real-world HSR exposure and injury outcomes. Eligible outcomes included injury risk and/or injury occurrence, operationalized as time-loss injuries, medical-attention injuries, specific injury types (e.g., non-contact muscle injuries, hamstring injuries), or injury rates where exposure denominators were available, provided that the study reported extractable associations between HSR-related exposure measures and injury outcomes.

Studies were excluded if they evaluated preventive strategies, intervention programmes, or experimental training manipulations in which sprinting or HSR exposure was prescribed as an intervention (e.g., “sprint training” or “HSR exposure programmes” intended to prevent injury), because such studies assessed intervention effects rather than associations between naturally occurring exposure and injury outcomes. Studies were also excluded if they focused on other football codes (e.g., American football, rugby) or non-11-a-side soccer variants as the primary sport (e.g., futsal, beach soccer), did not quantify HSR/sprinting exposure, or did not measure injury outcomes.

### 2.2 Information sources

Searches were conducted in PubMed (MEDLINE), Scopus, and Web of Science Core Collection, and each database was last searched on 20 January 2026. To identify additional eligible studies not captured by database indexing, the reference lists of all included studies and of relevant systematic reviews identified during screening were examined (backward citation searching), and forward citation searching of included studies was planned using Web of Science citation tracking on 20 January 2026.

### 2.3 Search strategy

Search strategies were developed to combine controlled vocabulary (where available) and free-text terms for (i) soccer/football, (ii) high-speed running/sprinting and external load monitoring, and (iii) injury outcomes. No date restrictions were applied, and no language restrictions were applied. Non-English records were eligible; however, no non-English full texts met inclusion criteria (or required full translation) after screening and full-text assessment, therefore no translated extractions were performed. When potentially eligible non-English full texts were identified, translation was planned using fluent speakers within the research team or professional translation where required to determine eligibility and enable extraction. The search strategy can be observed in Table 1.

**Table 1:** Search strategy.

**Domains**	**Search specificities**	**Search terms**
Soccer	Title, abstract and keywords (topic)	Soccer OR football OR footballers
		AND
High-speed running	Title, abstract and keywords (topic)	“High-speed running” OR “high speed running” OR “high-speed” OR “high speed” OR “HSR” OR sprint OR sprinting OR “maximal speed” OR “high-intensity running” OR “high-intensity” OR “high intensity” OR “external load” OR workload
		AND
Injury	Title, abstract and keywords (topic)	Injury OR injuries OR strain OR time-loss

### 2.4 Selection process

All records retrieved from the searches were exported into Zotero software for de-duplication, after which titles and abstracts were screened against eligibility criteria by two authors working independently. Full texts of records judged potentially eligible by either author were retrieved and assessed independently by the same two authors, with reasons for exclusion at the full-text stage recorded in a standardized decision log suitable for PRISMA flow reporting. Disagreements at any stage were resolved through discussion. If agreement could not be reached, an external expert acted as an arbitrator. Inter-rater agreement was quantified for title/abstract screening and full-text inclusion decisions, revealing an agreement of *k* = 0.91.

### 2.5 Data collection process

Two authors extracted data independently from each included study to minimize extraction error. Extracted fields were cross-checked and discrepancies were resolved by consensus. Data extraction was performed using a standardized form designed for this specific case.

### 2.6 Data items

The primary outcomes sought were injury risk and injury occurrence associated with HSR/sprint exposure, including (a) time-loss injury incidence or risk, (b) non-contact muscle injury incidence or risk, and (c) hamstring injury incidence or risk, as defined by each study. Secondary outcomes included injury rates expressed per exposure time, injury burden measures where reported, and injury outcomes stratified by context (training vs. match), season phase, sex, age group, or playing level. All results compatible with each outcome domain were sought when available (e.g., multiple time windows such as acute/chronic, multiple HSR thresholds, and multiple model specifications), and where multiple eligible estimates existed within a study, a prespecified hierarchy was applied prioritizing (i) adjusted models over unadjusted, (ii) clearly defined exposure windows aligned with acute (≤7 days) and chronic (≥21–28 days) constructs, and (iii) study-defined primary analyses over sensitivity or exploratory models. For screening and subsequent synthesis grouping, we grouped studies as addressing injury risk when the analysis estimated the probability/odds/hazard of subsequent injury from prior HSR/sprint exposure (i.e., prognostic or predictive modeling). Studies were classified as addressing injury occurrence when analyses were event- or context-based and descriptive/comparative (e.g., exposure in injury weeks vs. control weeks, or exposure in periods surrounding injury events) without an explicit prognostic model for future injury. If a study reported both an unadjusted injury-week vs. control comparison and an adjusted mixed-effects model estimating injury odds from prior 7-day HSR distance, we selected the adjusted mixed-effects model estimate for the main synthesis (while retaining the comparative estimate in the extraction table).

The following variables were extracted to support interpretation, subgrouping, and heterogeneity exploration: study design and context; sample size; participant demographics (age, sex), playing level, and season phase; injury definition and surveillance method; HSR/sprinting definitions (absolute thresholds, individualized thresholds, and device/processing specifications); exposure context (training vs. match), exposure windows (acute, chronic, acute:chronic constructs, rolling averages), and whether exposure was expressed as distance, time, counts, or peak values.

### 2.7 Study risk of bias assessment

Risk of bias in individual studies was assessed using the Quality In Prognosis Studies (QUIPS) tool (28) because our review focus on prognostic/risk factor associations between exposure and injury outcomes. Two authors assessed each study independently across QUIPS domains (study participation, study attrition, prognostic factor measurement, outcome measurement, study confounding, and statistical analysis and reporting), and domain-level judgments were summarized into an overall risk-of-bias judgment using prespecified decision rules emphasizing confounding control and measurement validity for HSR exposure and injury outcomes. Disagreements were resolved by consensus.

### 2.8 Synthesis methods and effect measures

Effect measures were extracted quantitatively from each included study wherever possible, prioritizing estimates from the most fully adjusted models that aligned with the study's primary analysis and with the review's exposure and outcome definitions. For dichotomous injury outcomes, extracted measures included odds ratios, risk ratios, and hazard ratios, and for rate-based outcomes (e.g., injuries per exposure time), incidence rate ratios were extracted when reported. Where multiple eligible effect estimates were available within a study for the same exposure–outcome pairing (e.g., multiple HSR thresholds, time windows, or model specifications), a single estimate was selected for the main synthesis using a prespecified hierarchy that prioritized adjusted over unadjusted models, clearly defined exposure windows corresponding to acute and chronic constructs, and study-defined primary analyses over exploratory or sensitivity analyses, to avoid double counting and reduce selective emphasis.

Studies were organized into synthesis groups based on conceptual comparability of (i) exposure construct (HSR vs. sprint; absolute vs. individualized thresholds; metric type such as distance, time, counts, or peaks), (ii) exposure context (training, match, or combined), (iii) exposure window (acute, chronic, and other prespecified rolling-average constructs), and (iv) injury outcome definition (all injuries, non-contact muscle injuries, hamstring injuries, or other predefined categories). The primary approach to synthesis was structured narrative synthesis supported by quantitative extraction, including standardized tabulation of study characteristics and results, and direction-of-effect summaries within each synthesis group. Where effect estimates were sufficiently comparable, results were presented side-by-side using consistent units and increments (e.g., per 100 m HSR distance, per 1 SD increase, or per prespecified workload change) when feasible, but no statistical pooling was undertaken because post-extraction assessment demonstrated substantial incompatibility in exposure definitions (absolute vs. individualized thresholds; differing speed bands), exposure windows and scaling (e.g., acute periods defined variably; differing unit increments), outcome definitions (time-loss vs. non-contact soft-tissue vs. specific diagnoses), and effect measures/model structures, which precluded defensible harmonization for meta-analysis. Accordingly, synthesis followed a structured narrative approach consistent with guidance for synthesis without meta-analysis. Heterogeneity was explored qualitatively by comparing patterns of association across prespecified subgroups (sex, age group, competitive level, exposure definition, exposure context, and season phase) and by examining how analytical decisions (covariate adjustment, handling of repeated measures, and injury definition) influenced findings.

To limit selection bias when multiple thresholds or windows were reported, we applied the prespecified hierarchy consistently and extracted all eligible estimates into evidence tables, but selected one estimate per exposure–outcome pairing for narrative direction-of-effect summaries (with sensitivity reporting when an alternative threshold/window materially altered interpretation). When a study reported multiple injury outcomes (e.g., all injuries and hamstring injuries), we treated each outcome as a separate synthesis group and avoided combining outcomes within a single direction-of-effect judgment.

## 3 Results

### 3.1 Study selection

**Figure 1:**
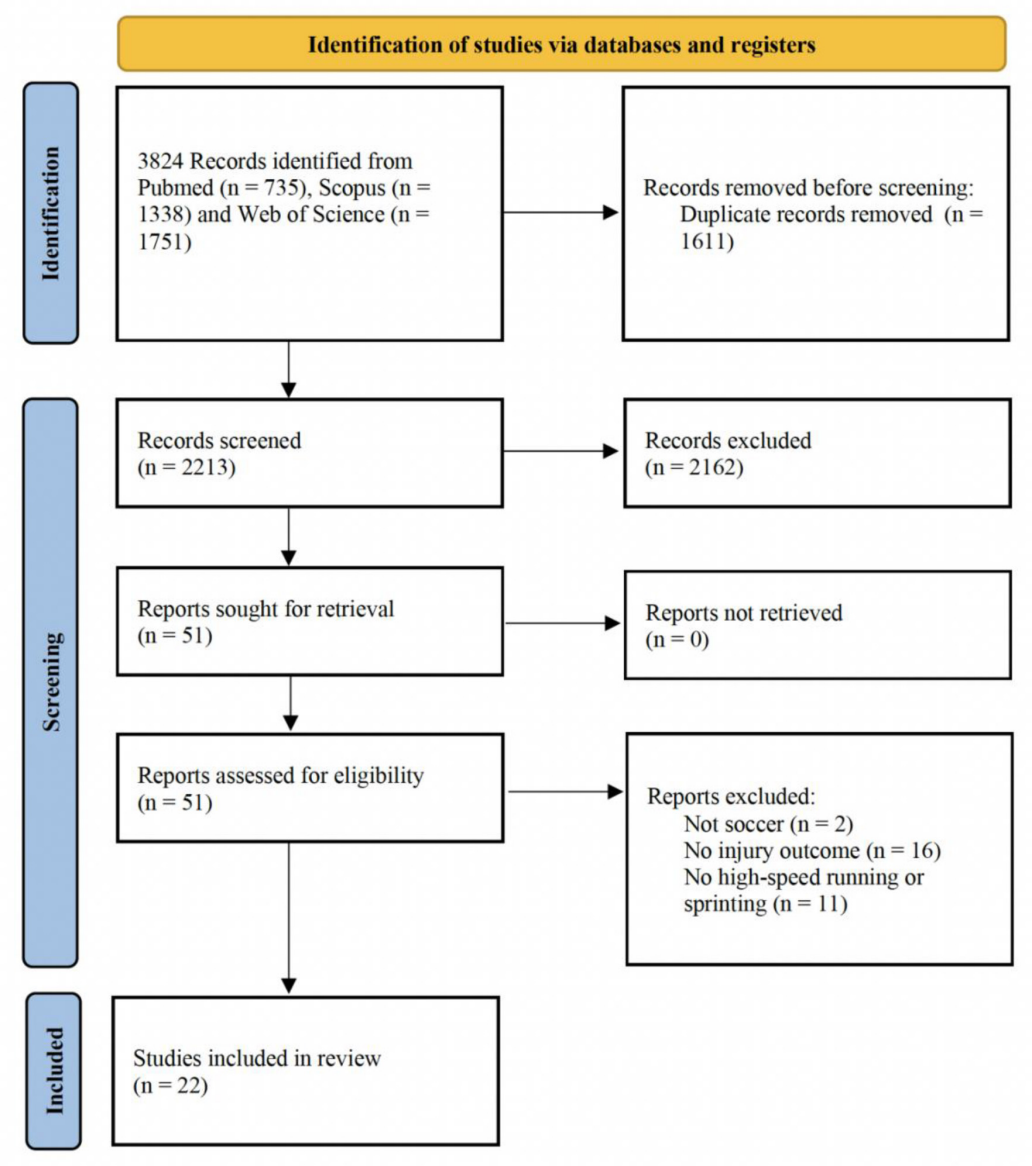
Prisma flow diagram.

Figure 1 summarizes the study selection process using the PRISMA 2020 flow diagram. Database searching identified 3,824 records from PubMed (*n* = 735), Scopus (*n* = 1,338), and Web of Science (*n* = 1,751). After removing 1,611 duplicates, 2,213 records were screened by title and abstract, and 2,162 were excluded. Full texts were sought for 51 reports, all of which were retrieved, and subsequently assessed for eligibility. Of these, 29 reports were excluded for predefined reasons [not soccer, *n* = 2 (29, 30); no injury outcome, *n* = 16 (31–46); no high-speed running or sprinting outcome, *n* = 11 (17, 47–56)], resulting in 22 studies included in the final review.

### 3.2 Study characteristics

Across the 22 included studies (Table 2), most were conducted in European contexts (16/22; 72.7%), with Spain the most frequently represented country/league (5/22; 22.7%), followed by England (4/22; 18.2%), and Portugal, Greece, and Australia (each 2/22; 9.1%). The predominant playing level was professional/elite football (including first team, reserve, and multi-club professional teams) (18/22; 81.8%), with fewer studies in youth academy (2/22; 9.1%), and single studies in semi-professional (1/22; 4.5%) and collegiate football (1/22; 4.5%). Most investigations were single-team/club (19/22; 86.4%), with three studies sampling multiple clubs/teams (3/22; 13.6%). Sample sizes ranged from 11 to 192 players, and most studies included ≤50 players (20/22; 90.9%). The 22 included studies comprised both injury risk (prognostic/predictive modeling; *n* = 14) and injury occurrence (event-based/descriptive/contextual; *n* = 8) designs, which are synthesized separately in Tables 5, 6.

**Table 2:** Methodological characteristics of the included studies.

**Study**	**Country/league**	**Playing level**	**Sample (players/teams)**	**Sex**	**Age (mean ±SD or range)**	**Follow-up/season phase**
Aiello et al. (73)	Italy/Serie A	Elite pro club; training and matches	46 players/1 team	Male	26.6 ± 4.3 y	3 seasons
Bacon et al. (66)	England (Norwich City FC Academy)	Pro youth academy (U18–U21); training and matches	41 players/1 team	Male (NR)	17.8 ± 1.1 y	2 seasons (2012–13; 2013–14; ~40 wk)
Barreira et al. (78)	Portugal/Liga I	Pro first team; training and matches	33 players/1 team	Male	NR	2 seasons (2021–22; 2022–23)
Bowen et al. (57)	England/Premier League Category 1 academy (U18–U21 Premier League)	Elite youth academy; training and matches	32 players/1 academy (52 player-seasons)	Male (NR)	17.3 ± 0.9 y	2 seasons (2013–14; 2014–15)
Bowen et al. (58)	England/Premier League	Professional first team; training and matches	33 players/1 club (61 player-seasons)	Male (NR)	25.4 ± 3.1 y	3 seasons (2014–15; 2015–16; 2016–17)
Enright et al. (61)	Europe/8 clubs competing in UEFA leagues (countries NR)	Professional soccer; in-season training and matches	192 players/8 teams (264 non-contact injuries)	Male (NR)	NR	2 seasons (2015–16; 2016–17); 28-day pre-injury window
Fousekis et al. (59)	Greece/Super League 1 and Super League 2	Professional men's soccer; training + matches	40 players/1 professional cohort (300 training sessions; 60 official matches)	Male	20.6 ± 1.6 y	2 seasons (2021–22; 2023–24)
Gregson et al. (72)	Qatar/Qatar Stars League	Professional soccer; competitive matches only	29 outfield players/1 club cohort (276 competitive matches)	Male (NR)	NR	2 seasons (2013–14; 2014–15)
Guitart et al. (71)	Spain/Elite club competing in Spanish + European leagues	Elite club; youth (U18/U19) + professional reserve team; training + matches	71 players/1 club (41 youth; 30 professional)	Male	U18: 16.01 ± 0.71 y; U19: 17.02 ± 0.70 y; Pro: 20.5 ± 5.45 y	1 season (2017–18)
Herazo-Sánchez et al. (76)	Colombia/professional club (league NR)	Professional soccer; training and matches	31 players/1 club; 145 training sessions + 26 matches monitored	Male	25.2 ± 4.8 y	1 season (2021–1)
Kim and Choi (60)	South Korea/collegiate football (university team); tournament + U-league matches	Collegiate football (soccer); match play (≥80 min) only	11 players/1 university team; 17 matches (143 player-match observations)	NR	≈22 y (injured 22.0 ± 1.67; non-injured 22.4 ± 1.14)	Jul 2019–Jan 2020; 17 matches; ACWR computed from week 4 onward
Lu et al. (70)	Australia/A-League (also Asian Champions League)	Professional male soccer; training and matches (GPS collected in training only)	45 players/1 team; 2 seasons; 39 non-contact injuries analyzed	Male	26.4 ± 5.1 y	2013/14 (14 w preseason + 32 w in-season) and 2014/15 (14 w preseason + 31 w in-season)
Malone et al. (16)	Portugal/Liga NOS	Elite professional soccer; training + match play	37 players/1 squad	Male	25 ± 3 y	2015–2016 season; 48 weeks (all training sessions + match play)
Massard et al. (69)	Australia/Tier 2 (two semi-professional clubs)	Semi-professional soccer; on-pitch training + matches	47 players/2 clubs (62 individual season observations; goalkeepers excluded)	NR	22.9 ± 4.1 y	2016 and 2017 seasons; preseason to final match (28–34 weeks)
Morgans et al. (74)	England/Premier League	Elite professional football (1 EPL club); training + matches	30 outfield players/1 team	Male	24.2 ± 6.1	2020/21 season (pre-season + competitive season); weeks −4 to −1 preceding injury
Nilsson et al. (75)	Sweden/Swedish professional football (3 clubs)	Male professional football; training + matches	25 players/3 teams (25 HSIs)	Male	26 ± 4	Four seasons; 7- and 14-day periods preceding injury vs. matched control periods (baseline 15–28 days pre-injury)
Nobari et al. (65)	Iran/Persian Gulf Pro League	Professional football; league + knockout tournament; natural grass	21 outfield players/1 team (2018–2019 season; 48 weeks; 200 training sessions; 44 matches)	NR	28.3 ± 3.8	Full 2018–2019 season (48 weeks); weekly analyses (congested vs. non-congested weeks)
Piłka et al. (79)	Poland/PKO BP Ekstraklasa	Professional male football club; training + matches (routine GPS monitoring)	36 outfield players/1 club (2 rounds: spring 2020–2021; fall 2021–2022; 1,064 microcycles)	Male	24.0 ± 5.3 y	2 competitive rounds (spring 2020–2021; fall 2021–2022); microcycle-based dataset
Raya-González et al. (67)	Spain/LaLiga	Professional male football; training + matches on natural grass (~100 × 70 m)	26 outfield players/1 team (2020–2021: 192 training sessions, 41 matches; 2021–2022: 229 sessions, 42 matches; 38 muscle-tendon injuries incl. 15 hamstring strains)	Male	28.8 ± 4.4 y	2 seasons (2020–2021 and 2021–2022)
Soler et al. (68)	Spain/LaLiga	Professional male football; routine monitoring during training + matches	41 players/1 team (2 seasons 2020–2021 and 2021–2022; 16 non-contact calf strain injuries in 10 players)	Male	28.2 ± 4.0 y	2 seasons; week prior to injury vs. control (mean of 6 prior weeks; week −1 excluded)
Suarez-Arrones et al. (62)	Spain/La Liga (reserve squad; UEFA Champions League club)	Professional reserve-squad soccer; outdoor natural grass; training + official matches	15 players/1 team	Male	18.6 ± 0.8 y	2018–2019 season; 10 consecutive competitive microcycles (10 training weeks + 10 official matches) + 4 prior weeks for initial ACWR
Tsilimigkras et al. (77)	Greece/Greek Super League	Professional male soccer club (Asteras Tripolis FC); training sessions + official matches	25 outfield players with first-time non-contact muscle injury; monitored across 3 seasons (2021–2024; 665 training sessions, 111 games)	Male	NR	3 seasons (2021–2024); 28-day pre-injury epoch vs. 28-day baseline epoch

NR, no reported; y, years old; ACWR, acute:chronic workload ratio.

Across the 22 included studies (Table 3), GPS-based wearable monitoring systems were used in almost all investigations (21/22; 95.5%). Definitions of HSR/sprinting were predominantly based on absolute speed thresholds (20/22; 90.9%). Exposure was most often quantified across both training and matches (19/22; 86.4%). Exposure windows were most commonly aggregated over weekly and/or 1–4-week rolling periods (15/22; 68.2%). Injuries were most often defined using time-loss criteria (13/22; 59.1%) and were primarily captured via medical staff/records (20/22; 90.9%). The most frequent outcome categories were soft-tissue/muscle-related injuries (e.g., hamstring/calf/lower-limb muscle-tendon injury outcomes) (14/22; 63.6%).

**Table 3:** Instruments and outcomes in the included studies.

**Study**	**Monitoring system**	**HSR/sprint definition**	**Exposure context**	**HSR/sprint metric(s)**	**Exposure window(s)**	**Injury definition**	**Injury surveillance source**	**Outcome category**
Aiello et al. (73)	Catapult Vector S7 GPS 10 Hz (+100 Hz IMU); OpenField 3.4.0; video (training fixed-camera; matches TV)	No fixed threshold; peak speed + %individual max (reported >25 km/h and >80% max)	Training + match (injury event)	Peak speed; %max; run distance; mean acc/dec	N/A (event-based; < 2 s)	Sudden-onset non-contact; identifiable inciting event; gradual-onset excluded	Team doctor + medical records; video analysis	Non-contact (hamstring, adductor, quadriceps, calf)
Bacon et al. (66)	StatSports Viper Pod GPS (sampling NR); same unit per player; worn upper back	HSR distance (m); threshold NR	Training + match (all sessions/matches)	TD (m); HSR (m); weekly avg; 2–4 w cumulative; load groups (±1 SD)	Weekly + rolling 2–4 w	Overuse (physio-diagnosed); incidence per 1,000 h on-leg exposure; severity by days missed	Club injury audit (physio)	Overuse
Barreira et al. (78)	NR	HSR and sprint distance; dist >80% and >90% max vel; acc/dec thresholds >2 and >3 m/s^2^; HSR/sprint speed NR	Weekly total; training vs. match	Weekly HSR; sprint; >80% and >90% dist; acc/dec meters >2; match acc/dec >3	Weekly (congested vs. non-congested)	Soft-tissue; rate and burden per 1,000 h (definition NR)	Team medical records (NR)	Soft-tissue
Bowen et al. (57)	StatSports Viper V.2 GPS 10 Hz (+100 Hz accelerometer); Viper software v2.1.3.0	HSD > 20 km/h (no separate sprint threshold reported)	Training + match (all on-pitch sessions and matches)	HSD distance (m) (plus TD, ACC, TL)	Weekly blocks (Mon–Sun); cumulative 1–4 wk; ACWR (acute = 1 wk; chronic = 4-wk rolling avg); *z*-score categories; chronic split high/low (median)	Time-loss (absence from future participation); severity: minimal/mild/ moderate/severe; contact vs. non-contact	Academy doctor + senior chartered physiotherapists; club database	Overall injury; contact vs. non-contact (also by type/site)
Bowen et al. (58)	StatSports Viper 2 GPS 10 Hz (+100 Hz accelerometer); Viper software v2.1.3.0; competitive matches via TRACAB video (files imported to Viper)	HSR 19.8–25.2 km/h; sprint >25.2 km/h	Training + match (training/friendly via GPS; competitive matches via video)	HSR distance (m); sprint distance (m)	Cumulative 1–4 w; ACWR uncoupled (acute = 1 w; chronic = previous 4-w avg); chronic split high/low (median); *z*-score categories	Time-loss injury; severity: minimal/mild/ moderate/severe; contact vs. non-contact	Club doctor + senior chartered physiotherapists	Overall injury; contact and non-contact injury
Enright et al. (61)	Catapult OptimEye S5 GPS (firmware 717); Openfield v1.14; min effort 0.4 s; device assigned per player; satellites 14 ± 2 (HDOP 0.77 ± 0.03)	HSD > 5.5 m/s; sprint > 7.0 m/s (absolute thresholds)	Training + match (all on-pitch sessions and matches during in-season)	HSD distance; sprint distance (also total distance)	28-day window pre-injury; accumulated 1–4 w loads (7-day blocks aligned to injury date); ACWR coupled and uncoupled (1:3; 1:4) + EWMA; acute = avg 7 days pre-injury	Non-contact time-loss injury; tissue type (muscle/tendon/ligament) confirmed by ultrasound; first-time injuries only; severity by days missed (minimal/mild/ moderate/severe)	Club medical procedures (Munich Consensus); diagnosed by medical doctors and qualified physiotherapists; central database	Non-contact injuries by tissue type (muscle/tendon/ ligament) and severity
Fousekis et al. (59)	Catapult Vector S7 GPS (10 Hz); same unit per player (processing software NR)	Zones: HSR 14.4–19.8 km/h; very HSR 19.8–25.0 km/h; sprint >25.0 km/h; ACC/DEC >2.5 m/s^2^	Training + match (full participation only; GK excluded)	Distance in speed zones (TD, HSR, very HSR, sprint); ACC/DEC counts or distance	Weekly accumulated loads; rolling ACWR (acute = injury week; chronic = mean of prior 1–4 weeks); 4-week pre-injury window (W-4 to W-1) matched to non-injury reference periods	Non-contact time-loss injury: physical complaint during training/match without direct contact causing ≥ 1 missed session/match; severity: minimal/mild/ moderate/severe	Team medical doctor diagnosis after medical examination (contact injuries excluded)	Non-contact time-loss injuries (lower-limb muscle injury distribution reported)
Gregson et al. (72)	Multiple-camera player tracking system (STATS, London, England); match physical performance data	HSR distance > 19.8–25.2 km/h; sprint distance > 25.2 km/h; additional categories: running > 14.4–19.8 km/h	Match only (competitive matches)	Distance in speed zones (incl. HSR and sprint); sprint counts (leading vs. explosive sprints)	Pre-injury periods: 1-min and 5-min preceding injury; additional description of final 5 s pre-injury; “habitual” normative profile derived from season match data	Match muscle injuries diagnosed and recorded by team physician; injury characteristics (type/location/diagnosis; occasion; minute) recorded per consensus statement	Team physician injury card; injury incident cross-referenced to tracking system availability (cases excluded if injury timing not discernible)	Match muscle injury occurrence
Guitart et al. (71)	WIMU PRO (RealtrackSystems, Spain) GPS/IMU (sampling frequency NR); SPRO software v927 (RAW export)	HSR distance > 21 km/h; HMLD: metabolic power > 25.5 W/kg (incl. distance > 21 km/h and high ACC/DEC activities)	Training + match (incl. preseason; stratified by MD and days relative to match)	GPS volume metrics: HSR (m), HMLD (m), total distance (m), player load (AU) (also per-minute intensities)	Season-long exposure; stratified by day relative to match (MD, −4 MD to −1 MD, + 1 MD to + 2 MD) and preseason	UEFA consensus definitions; time-loss (miss ≥ next session/match) vs. no-time-loss (medical attention only); lower-limb muscle injuries coded via OSICS-10 (-M–); recurrence defined within 2 months	Team doctor diagnosis (all diagnoses by same doctor); exposure time recorded by technical staff	Lower-limb muscle injuries (TL and NTL)
Herazo-Sánchez et al. (76)	Catapult OptimEye S5 GPS 10 Hz; Catapult software (OptimEye S5); data collected per standard GPS guidelines	HSR: distance >20 km/h; efforts 20–25 km/h; sprint: distance >25 km/h and sprint efforts >25 km/h	Training + match	Duration; total distance; HSR distance >20 km/h; efforts 20–25 km/h; distance >25 km/h; sprint efforts >25 km/h; accelerations >1 m/s^2^; decelerations < -1 m/s^2^	Season-long monitoring; specific acute/chronic or rolling window NR	Non-contact hamstring injury; diagnoses coded per FIFA consensus; hamstring tear confirmed by MRI (club doctor)	Club medical staff; club doctor (MRI-based confirmation); injury data shared via performance analyst to researchers	Non-contact hamstring injuries
Kim & Choi (60)	PlayerTek GPS (Catapult Innovations) 10 Hz; PlayerTek software; unit placed at T2–T6; matches only (≥80 min); warm-up and half-time excluded	High-intensity running ≥18 km/h; sprint ≥21 km/h (distance and bouts). Additional thresholds: work:rest based on ≥4.0 vs. ≤ 3.9 km/h; accelerations ≥ 2.78 m/s^2^; decelerations ≤ −2.78 m/s^2^	Match only	Total distance; high-intensity distance (≥18 km/h); sprint distance (≥21 km/h); sprint bouts; work:rest ratio; acceleration and deceleration bouts; ACWR for each variable	Weekly cumulative loads; ACWR (acute = 1 week; chronic = 4-week mean) from week 4; ACWR split into quartiles for logistic regression	Sports injury defined as any musculoskeletal pain/discomfort; analysis restricted to non-contact soft-tissue injuries in low back and lower extremity (hip, thigh, knee, crura, ankle, foot)	Daily injury report (IOC-based) recorded throughout data collection	Non-contact lower-extremity (and low back) injuries
Lu et al. (70)	SPI HPU GPSports GPS 15 Hz (10 Hz interpolated to 15 Hz) + 100 Hz 16G triaxial accelerometer; training sessions only (matches not tracked by GPS due to FIFA regulations)	High-speed running > 14.5 km/h; very-high-speed running > 20 km/h (low-speed < 14.4 km/h)	Training only for GPS-derived HSR/VHSR (exposure and s-RPE included training + matches)	Total distance; distance by speed zones (LSR/HSR/VHSR); mean speed; bodyload (AUs)	4-week workload profile per injury (3, 2, 1 weeks prior and injury week); expressed as absolute weekly cumulative, week-to-week change, and relative to season mean; ACWR acute = week prior; chronic = mean of prior 3 weeks (injury week excluded)	Time-loss injury: any physical complaint from training/match resulting in time loss (Fuller et al.); non-contact injuries included; contact injuries excluded	Club injury records collated by sport science/conditioning staff per governing national body definition	Non-contact time-loss injuries (all types)
Malone et al. (16)	GPS (STATSports Viper, Northern Ireland); 10 Hz; worn between scapulae; Viper software v3.2; sRPE recorded 30 min post-session via bespoke app	HSR >14.4 km/h; sprint >19.8 km/h (absolute)	Training + matches	HSR distance; sprint distance; internal load via sRPE (AU)	Chronic load: 21-day average; acute load: 3-day; soccer-specific ACWR (3:21 day) + weekly change in load	Time-loss > 24 h: unable to take full part in planned training/match activities (Brooks definition); severity (low/moderate/high); injury type/body site/mechanism recorded	Bespoke club injury database (medical/performance staff)	Lower-limb time-loss injuries (focus on non-contact lower-limb injury risk)
Massard et al. (69)	GPS (MinimaxX S4, Catapult Sports, Australia); 10 Hz; Catapult Sprint v5.1.7 (Intelligent Motion Filter, min effort 0.2 s); additional signal-quality filtering; missing data estimated per protocol	Absolute HSR ≥19.8 km/h (5.5 m/s); individualized HSR threshold based on 30–15 IFT final speed (vIFT)	Training + matches	HSR distance per session (absolute and individualized thresholds)	Cumulative loads via EWMA (acute 7 d; chronic described as 28 d in abstract; chronic load formula reported with 21 d decay and 7 d offset)	Time-loss (Fuller): unable to take full part in future training or match play; only non-contact lower-limb time-loss injuries included (incl. reinjuries)	Onsite chartered physiotherapist at each club (attending all sessions and matches)	Non-contact lower-limb time-loss injuries
Morgans et al. (74)	STATSports Apex Pod v4.03 (18 Hz GNSS; players wore same unit); processed in Apex software v4.3.8 (10 Hz); dwell time 0.5 s (HIR) and 1.0 s (sprint); mean satellites 21 ± 3; HDOP 0.9–1.3	High-intensity distance: 5.5–7.0 m·s−1; Sprint distance: >7.0 m·s−1 (absolute thresholds)	Both (all training sessions and matches)	Total distance, high-intensity distance, sprint distance (absolute m and relative m·min−1)	4-week period preceding injury (weeks −4, −3, −2, −1) + season totals (2020/21)	Time-loss injury surveillance (Fuller et al.); injury causing modified training and/or missed training session(s) or match(es); only hamstring injuries analyzed	Club medical department; physiotherapist + head doctor; daily availability/injury records (standardized format)	Hamstring time-loss injuries
Nilsson et al. (75)	Catapult Vector S7 (10 Hz GPS; units placed between shoulders; same unit per player); datasets verified (mean satellites >11; mean HDOP < 1.2); processing software NR	HSR distance: >19.8 km·h−1; maximal sprint distance: >29.8 km·h−1 (absolute thresholds)	Both (all outfield training sessions and matches)	Total distance; HSR distance; maximal sprint distance; maximal velocity; very intense accelerations (>3.0 m·s−2) and decelerations (< -3.0 m·s−2)	7-day and 14-day windows preceding injury (incl. injury day) vs. equally long control windows immediately preceding; baseline 15–28 days pre-injury	Hamstring strain injury (HSI) defined as any absence from future football participation (time-loss); prospectively recorded after each training/match; reinjuries excluded	Teams' medical staff; standard injury form (per Waldén et al.)	Hamstring strain (time-loss) injuries
Nobari et al. (65)	GPSPORTS SPI High Performance Unit (HPU): 15 Hz GPS; 100 Hz accelerometer; 50 Hz magnetometer; manufacturer belt; default SPI IQ Absolutes settings	HSD: 18–23 km·h−1; sprint distance (SPD) + repeated sprints (RS): thresholds NR (device default settings)	Both (each training and match session)	Weekly total distance (TD), high-speed distance (HSD), sprint distance (SPD), meters of repeated sprints (RS)	Weekly load across season; dichotomised high-load vs. low-load weeks (cut-offs defined from team averages)	Non-contact injuries (no contact with foreign material/athletes) recorded across season; time-loss/medical-attention criteria NR	Season injury recording (source NR; likely club staff/records)	All non-contact injuries
Piłka et al. (79)	Catapult Vector S7 10-Hz GPS (4 GHz) + IMU (100 Hz accel/gyro/magnetometer); OpenField; same unit per player	HSR: 19.8–25.2 km/h; Sprint: >25.2 km/h; Acc/Dec: >2–3 m/s^2^	Both (training microcycle + match day; separate aggregates)	HSR & sprint distances; acceleration/deceleration metrics; microcycle totals/ratios	Microcycle aggregates; lagged 1–3 microcycles; cumulative season-to-date and ACWR-type features (e.g., PlayerLoad current vs. previous 3 microcycles)	Non-contact lower body injuries (medical report); binary label per microcycle (injury vs. no injury)	Player report to medical team; club medical reports	Non-contact lower-body musculoskeletal injuries
Raya-González et al. (67)	WIMU PRO 10-Hz GPS (RealTrack Systems); SPRO software; same unit per player	HIR 18–21 km/h; HSR 21–24 km/h; SR >24 km/h; sprints: actions ≥25 km/h; LACC/LDEC 2–4 m/s^2^; HACC/HDEC >4 m/s^2^	Both (weekly cumulative training + match exposure)	Distances in HIR/HSR/SR; number of sprints; acceleration/deceleration distances	Week prior to injury (7 d) vs. mean of 6 previous weeks (weeks−6 to−1; week−1 excluded)	Non-contact muscle-tendon injuries (IOC classification); time-loss (missed subsequent session); severity by days absent	Team medical staff; standardized injury form; examinations/imaging as required	Non-contact muscle-tendon injuries (hamstring strain sub-analysis)
Soler et al. (68)	10-Hz GPS-accelerometer (WIMU PRO, RealTrack Systems); SPRO software; activated ~15 min pre-session; same unit per player	HIR 18.0–20.9 km/h; SR 21.0–23.9 km/h; high SR ≥24.0 km/h; sprint actions >21 km/h for ≥1 s; LACC/LDEC 2.0–3.9 m/s^2^; HACC/HDEC ≥ 4.0 m/s^2^	Both (weekly cumulative training + match exposure)	TD; distances in HIR/SR/high SR; sprint actions; LACC/HACC/ LDEC/HDEC distances; HMLD; exposure time (min)	Week before injury (7 d, excluding injury day) vs. mean of 6 prior weeks excluding week immediately before injury	Non-contact calf muscle strain (IOC classification); time-loss (missed following session); severity by days absent	Team medical staff; standardized form; clinical exam and imaging as needed	Non-contact calf muscle strain injuries
Suarez-Arrones et al. (62)	GPS (EVO; GPSports Systems, Canberra, Australia); 10 Hz; worn between scapulae; manufacturer processing/software	Speed zones: MSD > 14.4 km/h; HSD > 18.0 km/h; VHSD > 21.0 km/h; sprint > 24.0 km/h (absolute)	Training + matches	TD; MSD; HSD; VHSD; sprint distance (weekly totals for ACWR)	Uncoupled ACWR: acute = current week; chronic = 4-week rolling average (4 weeks prior to first microcycle also monitored); danger zone ACWR > 1.5	Time-loss (Fuller): absence from future football participation; severity categories (minimal/mild/ moderate/severe); contact vs. non-contact recorded	Medical practitioner (routine club injury surveillance/records)	All time-loss injuries
Tsilimigkras et al. (77)	Polar Team Pro 10-Hz GPS + accelerometer + 3D gyroscope + heart-rate belt/vest; Polar web-based software	HSR distance: speed > 19.8 km/h; sprint distance: speed > 25.2 km/h; accelerations/decelerations count > 2 m/s^2^ (sprint count threshold NR)	Both (training sessions + official matches)	DTOT; HSR distance; sprint distance; number of sprints; accelerations/decelerations; plus HR zones (80%−90%, 90%−100%) and Polar training load score	28-day epoch pre-injury (DEV max vs. average; modified ACWR acute 7 d vs. chronic 4 w excluding last week) vs. 28-day non-injury baseline epoch	First-time (within ≥12 mo), non-contact muscle injury (hamstring/adductor/ calf/quads); time-loss NR	NR (club data; injuries identified per study inclusion criteria)	Non-contact muscle injuries

NR, non-reported; HSR, high-speed running; GPS, global positioning system; TD, total distance; SR, sprint running; ACWR, acute-chronic workload ratio; HIR, high-intensity running; HMLD, high metabolic load distance.

### 3.3 Risk of bias

Across the 22 included studies, study participation was most frequently judged at moderate risk of bias (21/22; 95.5%), with high risk in 1/22 (4.5%). For study attrition, ratings were predominantly moderate (15/22; 68.2%), followed by high (6/22; 27.3%) and low (1/22; 4.5%). For prognostic factor measurement (HSR exposure), classifications were more evenly distributed, with low risk in 10/22 (45.5%) and moderate risk in 9/22 (40.9%), while low–moderate, moderate–high, and high risk were each observed in 1/22 (4.5% each). For outcome measurement (injury), most studies were rated low risk (14/22; 63.6%), with moderate risk in 5/22 (22.7%) and high risk in 3/22 (13.6%). Study confounding was the most consistently problematic domain, rated high risk in 19/22 (86.4%) and moderate risk in 3/22 (13.6%). For statistical analysis and reporting, most studies were judged high risk (15/22; 68.2%), followed by moderate (6/22; 27.3%) and low (1/22; 4.5%). Overall, the overall risk of bias was classified as high in 20/22 studies (90.9%) and moderate in 2/22 (9.1%). Table 4 shows the risk of bias assessment.

**Table 4:** Risk of bias of the included studies.

**Study**	**Study participation**	**Study attrition**	**Prognostic factor measurement (HSR exposure)**	**Outcome measurement (injury)**	**Study confounding**	**Statistical analysis and reporting**	**Overall RoB**
Aiello et al. (73)	Moderate	High	Moderate	Moderate	High	Moderate	High
Bacon et al. (66)	Moderate	Moderate	Moderate	Moderate	High	High	High
Barreira et al. (78)	Moderate	High	High	High	High	High	High
Bowen et al. (57)	Moderate	Moderate	Moderate	Low	High	High	High
Bowen et al. (58)	Moderate	Moderate	Moderate	Low	High	High	High
Enright et al. (61)	Moderate	High	Moderate	Low	High	High	High
Fousekis et al. (59)	Moderate	Moderate	Low	Low	High	Moderate	High
Gregson et al. (72)	Moderate	Moderate	Low	Low	Moderate	Moderate	Moderate
Guitart et al. (71)	Moderate	Moderate	Low	Low	High	High	High
Herazo-Sánchez et al. (76)	Moderate	Moderate	Moderate	Low	High	High	High
Kim and Choi (60)	Moderate	Moderate	Moderate	High	High	High	High
Lu et al. (70)	Moderate	High	Moderate	Moderate	High	High	High
Malone et al. (16)	Moderate	Moderate	Low	Low	High	Moderate	High
Massard et al. (69)	Moderate	Moderate	Low	Low	Moderate	Low	Moderate
Morgans et al. (74)	Moderate	High	Low	Low	High	High	High
Nilsson et al. (75)	Moderate	Moderate	Moderate	Moderate	High	Moderate	High
Nobari et al. (65)	Moderate	Moderate	Moderate	High	High	High	High
Piłka et al. (79)	Moderate	Moderate	Low	Low	Moderate	High	High
Raya-González et al. (67)	Moderate	Moderate	Low	Low	High	High	High
Soler et al. (68)	Moderate	Moderate	Low	Low	High	High	High
Suarez-Arrones et al. (62)	Moderate	High	Moderate	Low	High	Moderate	High
Tsilimigkras et al. (77)	High	Low	Low	Moderate	High	High	High

HSR, high-speed running; RoB, risk of bias.

### 3.4 Results of individual studies

Table 5 summarizes the 14 included studies that evaluated injury risk (i.e., probability/odds/hazard of subsequent injury from prior exposure, thus prognostic/predictive models) using inferential or predictive modeling (i.e., estimates of subsequent injury probability/odds/hazard from prior HSR/sprint exposure). Across these models, the most frequently reported higher-risk signals were linked to relative-change constructs (e.g., ACWR/EWMA-type contrasts or large week-to-week changes), whereas associations with absolute weekly HSR volume were more often null, small, or non-monotonic within “typical” load ranges. Where effect sizes were reported for spike/contrast approaches, associations sometimes reached multi-fold magnitudes (e.g., RR values in the ~5–7 range under “very high” ACWR conditions in low chronic-load strata), but estimates varied by measure, windowing, and covariate handling. Outcomes were typically time-loss or non-contact soft-tissue injuries, although definitions and injury phenotypes varied across studies.

**Table 5:** Summary of individual studies focusing on workload–injury association models.

**Study**	**Primary results**	**Main finding**
Bacon et al. (66)	Linear regression: HSR groups not significant predictor of overuse injury incidence: *F*(1, 39) = 1.003, *p* = 0.323, *R*^2^ = 0.025. Incidence (per 1,000 h): HSR low 11.29; normal 9.75; high 6.08. Cumulative-load analysis (2-week window; high vs. normal): OR 0.580 (95% CI 0.330–1.021), *p* = 0.059.	HSR (“intensity”) did not significantly predict overuse injury incidence; findings did not indicate increased risk at higher HSR loads and suggested a near-significant trend toward lower odds of overuse injury with high 2-week HSR.
Bowen et al. (57)	Non-contact injury: moderate-high 4-week HSD (3,502–5,123 m) showed increased risk vs. other HSD categories: RR = 2.14 (95% CI 1.31–3.50), *p* = 0.003 (binary logistic regression; RR from exposure counts).	Moderate-high accumulated HSD was associated with increased injury risk; risk also increased under “spike” conditions (high ACWR combined with low chronic exposure) across multiple running-load metrics.
Bowen et al. (58)	Non-contact injury (low chronic decelerations < 1,731): very high ACWR DEC (>2.32) associated with higher risk: RR = 6.58, *p* < 0.01 (CI not reported) (binary logistic regression; RR from exposure counts).	ACWR spikes approaching/exceeding 2.0—particularly when chronic loads were low—were associated with ~5–7 × higher non-contact injury rates for several running-load metrics. However, very high acute HSD and sprint distance were not significantly associated in their analyses.
Enright et al. (61)	No differences in HSD workload by tissue type: EWMA ACWR HSD (muscle vs. tendon vs. ligament) ANOVA *F* = 0.17, *p* = 0.898; no significant correlations between workload variables and injury severity (*p* > 0.05).	Across accumulated weekly loads and multiple ACWR methods, TD/HSD/sprint distance metrics did not differentiate injury tissue type or severity. Distance-based load measures and ACWR alone may be insufficiently sensitive and suggest incorporating broader mechanical/physiological load measures and contextual factors.
Fousekis et al. (59)	ACWR DSR15–20 (week 3 prior to injury, W3): OR = 3104.00 (95% CI 12.60–762,252.51), *p* = 0.004, FDR-adjusted *p* = 0.016; AUC = 0.811 (95% CI 0.675–0.947) (binomial logistic regression + ROC). Robust sprinting signal: ACWR DSR > 25 significant across W1-W4 (e.g., W3 OR = 9.55 [1.69–53.92], *p* = 0.011, FDR-adjusted *p* = 0.024; AUC = 0.735). After FDR correction, only DSR15–20 and DSR > 25 models remained significant; TD and DSR20–25 were not significant; ACC/DEC associations did not retain significance after FDR.	Across four pre-injury weeks, ACWR for moderate-speed running (15–20 km/h) showed the highest discriminative performance (AUC ~0.75–0.81), and sprinting ACWR (>25 km/h) was also consistently associated with injury. These are workload-ratio signals for non-contact injury risk, while total distance and 20–25 km/h running were weaker predictors; ACC/DEC effects were not robust after multiple-comparison control.
Herazo-Sánchez et al. (76)	No association: load indicators showed no significant correlation with hamstring injury; logistic regression model non-significant (*p* > 0.05) and no predictor retained.	In a single-team season with only two events, none of the monitored external-load variables explained the incidence of non-contact hamstring injury.
Kim & Choi (60)	Highest ACWR quartiles increased injury odds for: total distance (ACWR 1.060–1.254: OR 8.80, 90% CI 1.35–57.42, *p* = 0.02); high-intensity distance (1.142–1.496: OR 7.50, 1.31–43.03, *p* = 0.02); sprint bouts (1.226–1.636: OR 7.50, 1.30–43.02, *p* = 0.02). Sprint distance showed lower odds at low–moderate ACWR (0.842–1.011: OR 0.10, 0.01–0.65, *p* = 0.01). Work-to-rest and accel/decel ACWRs were not significant.	Higher relative match-load changes (ACWR) in total distance, high-intensity running, and sprint bouts are associated with greater non-contact injury odds.
Lu et al. (70)	Compared with injury week, exposure and s-RPE workload were higher in weeks 3, 2, 1 pre-injury (*p* = 0.04/0.03/0.01 and *p* = 0.03/0.01/ < 0.01), and were ~114% of season mean pre-injury. However, the 114% “threshold” had low sensitivity: exposure 25.6% (20.2–33.5), specificity 73.9% (72.6–78.2); s-RPE sensitivity 16.3% (12.6–24.9), specificity 79.9% (70.3–86.1).	No distinct workload profile reliably predicted injury; the main signal was sustained higher exposure and s-RPE in the weeks leading into injury, but predictive utility was poor (low sensitivity).
Malone et al. (16)	U-shaped weekly exposure: moderate weekly HSR 701–750 m was protective vs. low HSR (≤674 m): OR 0.12 (90%CI 0.08–0.94); moderate weekly SR 201–350 m protective vs. low SR (≤165 m): OR 0.54 (0.41–0.85). Large week-to-week increases increased odds: HSR change 351–455 m: OR 3.02 (2.03–5.18); SR change 75–105 m: OR 6.12 (4.66–8.29) (vs. smallest-change references).	Injury risk increased with rapid “spikes” (large week-to-week changes) and with high ACWR (e.g., HSR ACWR > 1.25; SR ACWR > 1.35). Higher chronic load (≥2,584 AU) and better aerobic fitness (30–15VIFT) appeared to attenuate risk at higher HSR/SR exposures (i.e., improved tolerance to high-speed demands).
Massard et al. (69)	Largest fixed effect: training sessions showed lower odds vs. matches in both models: OR 0.28 (95%CI 0.17–0.44) (individualized model; similar in absolute model). Across the central 80th percentile of observed acute/chronic HSR loads, predicted injury risk and CIs stayed close to baseline (≈ within ~1%); extremes highly uncertain due to sparse observations.	Using individualized vs. absolute HSR thresholds did not change the interpretation of load–injury models; within typical load ranges, HSR loads had negligible association with injury risk, while contextual factors (match vs. training; coach) explained more variation.
Nobari et al. (65)	High- vs. low-load weeks: increased injury odds and risk for all variables (OR and RR; 95% CI). SPD: OR 6.9 (1.3–35.5); RR 3.7 (1.0–14.6). HSD: OR 4.6 (1.3–16.3); RR 2.6 (1.1–6.0). TD: OR 4.1 (1.2–14.1); RR 2.4 (1.1–5.2). RS: OR 4.3 (1.0–18.2); RR 2.7 (0.9–7.8).	Mean injuries/week were higher in high-load vs. low-load weeks for TD, HSD, SPD and RS (all *p* < 0.05). High weekly sprint workloads (particularly SPD/HSD) are associated with increased non-contact injury risk.
Piłka et al. (79)	Classification performance (test set): XGBoost (best approach, all features + rules): accuracy 90.0%, precision 92.0%, Recall 97.6%, F1 94.7. Rule-based model: accuracy 0.76, precision 0.53, recall 0.58, F1 0.53. fuzzy rule-based: accuracy 0.84, precision 0.16, recall 0.23, F1 0.19.	ML (XGBoost) was the most promising for predicting upcoming-microcycle injury risk; key influential predictors included training time 2 weeks prior, accelerations/decelerations, and HSR meters in the current microcycle, aligning with coaches' expectations.
Suarez-Arrones et al. (62)	Spikes in ACWR > 1.5 occurred repeatedly in non-injured players: 13 uninjured players exceeded ACWR > 1.5 at least once; counts by metric reported (TD: 1 player; MSD: 2; HSD: 2; VHSD: 2). The 2 injuries occurred when ACWR values in the 4 microcycles prior were < 1.5 (with 1 non-contact muscle strain; 1 concussion).	Very low injury count, ACWR “spikes” (threshold > 1.5) were dissociated from injury occurrence; spike thresholds were frequently exceeded without injury, while injuries occurred without exceeding the spike criterion.
Tsilimigkras et al. (77)	Model performance (injury vs. baseline epochs): SVM (RBF kernel) using combined feature set achieved accuracy 0.78 (permutation *p* < 0.01), sensitivity 0.73, specificity 0.85. Most prominent predictors included DEV in number of sprints, TL score, and time in HR zone 90%−100%; cumulative load predictors included total distance, HSR, sprint distance, and TL score.	A multivariate ML approach leveraging both acute variability (DEV/ACWR-derived) and cumulative load features showed moderate-to-good discrimination of injury vs. non-injury epochs; sprint-related and high-cardiovascular-load exposures featured prominently among influential predictors.

NR, not reported; HSR, high-speed running; HSD, high-speed distance; TD, total distance; SR/SPD, sprint running/sprint distance; ACWR, acute:chronic workload ratio; EWMA, exponentially weighted moving average; AUC, area under the receiver operating characteristic curve; RR/OR/HR/IRR, risk/odds/hazard/incidence-rate ratio; CI, confidence interval; IMU, inertial measurement unit; GNSS/GPS, global navigation satellite system/global positioning system; HIR, high-intensity running; HMLD, high metabolic load distance; ACC/DEC, accelerations/decelerations.

Table 6 summarizes the eight studies that examined injury occurrence (i.e., exposures surrounding the injury event) in relation to HSR/sprint exposure using event- or context-focused designs rather than primary multi-week risk modeling. These studies are therefore reported as occurrence-context evidence (what exposures surrounded injury events). Across occurrence designs, findings commonly indicated that some injuries occurred during or immediately after high-velocity actions (e.g., many hamstring injuries during >25 km·h^−1^ actions and/or >80% maximal speed), but the inferential target differs from prognostic modeling because exposure is measured around the event rather than as a predictor window for future injury. Several occurrence studies also used injury-week vs. baseline comparisons to describe unusually elevated external-load profiles preceding specific injury phenotypes, but these contrasts should be interpreted as descriptive markers of context rather than as transportable thresholds for prediction.

**Table 6:** Summary of individual studies focusing on injury occurrence, timing, and context.

**Study**	**Primary results**	**Main finding**
Aiello et al. (73)	16/17 hamstring injuries occurred while accelerating up to or decelerating from >25 km/h; relative-speed distribution: 5 at 70%−80%, 7 at 80%−90%, 4 at >90% of maximal speed.	Hamstring injuries predominantly occurred during high-speed linear running (>25 km/h, typically >80% maximal speed), suggesting the need for preparation/robustness for near-maximal running exposures rather than simply restricting maximal-speed running.
Barreira et al. (78)	Congested vs. noncongested: injury rate 3.9 vs. 3.2 injuries/1000 h; injury burden 71.8 vs. 60.5 days/1,000 h; no significant differences reported.	Congested scheduling showed no effect on soft-tissue injury rate or burden. Weekly training HSR/sprint outputs were higher in non-congested weeks, whereas matches in congested periods registered higher external loads (including accelerations/decelerations >3 m/s^2^).
Gregson et al. (72)	1-min pre-injury period: sprinting distance (+2 within-player SD = +11 m) increased odds of muscle injury: OR = 1.22 (95% CI 1.12–1.33) (conditional fixed-effects logistic regression). 5-min pre-injury period: running, HSR, and sprinting distances were all reported as trivial associations. Sprint-type counts (1-min pre-injury): leading sprints RR = 1.16 (95% CI 1.06–1.26) and explosive sprints RR = 1.09 (95% CI 1.00–1.19), but both were classified as trivial.	Short, high sprinting exposure immediately preceding injury (1-min window) showed a harmful association with muscle injury occurrence, whereas 5-min windows were less sensitive.
Guitart et al. (71)	Injury frequency: 34 muscle injury episodes (19 TL; 15 NTL). Incidence per exposure time: overall 2.57 injuries/10^3^ h; highest on MD 4.55/10^3^ h, followed by −3 MD 4.07/10^3^ h. Incidence per GPS load metrics peaked on −3 MD: IRHMLD 0.41 per 10^5^ m; IRHSR 1.59 per 10^5^ m; IRPL 5.06 per 10^5^ AU; IRTD 6.61 per 10^7^ m. By position, midfielders had the highest incidence across measures (followed by forwards).	Both external loads and muscle injury incidence were highest on match day and 3 days pre-match, and interpret the data as consistent with a direct proportionality between higher external load and higher muscle-injury incidence. Expressing injury incidence by GPS-derived load measures (HMLD/HSR/PL/TD), not only exposure time, may better characterize injury patterns; midfielders showed the highest injury incidence across measures.
Morgans et al. (74)	Only statistically significant difference reported: sprint distance per minute in week −1 was higher in injured vs. uninjured: 2.31 ± 1.13 vs. 1.53 ± 0.86 m/min; p=0.038; ES=-0.89. No significant differences for absolute or relative total distance or high-intensity distance across weeks −4 to −1.	Absolute weekly totals were broadly similar between groups. Only meaningful signal was an increase in relativized sprint intensity (m/min) in the week immediately preceding hamstring injury, suggesting potential value of relative intensity measures over absolute weekly volume for short-horizon pre-injury.
Nilsson et al. (75)	Linear mixed model: no statistically significant differences in any TL variable between pre-injury and control periods (e.g., HSRd *p* = 0.31 (7 d) and *p* = 0.61 (14 d); MSd *p* = 0.90 (7 d) and *p* = 0.41 (14 d); all *p* > 0.05). Group-level NAP indicated “no difference” overall (range 0.45–0.54).	Large interindividual variability in short-term TL patterns (some players higher, others lower pre-injury vs. control), but no consistent group-level pattern. Short-term external TL measures alone may have limited value for predicting HSI risk at group level and should be interpreted with contextual/multifactorial factors.
Raya-González et al. (67)	Within-player paired *t*-tests (injury week vs. control). All workload parameters higher in injury week (Δ% +16.08 to +36.64; *p* < 0.001 to 0.012). Example (all muscle-tendon injuries): SR distance 263.19 ± 164.01 vs. 331.39 ± 163.74 m (Δ% +25.92; *p* < 0.001; ES 0.61); sprint count 17.97 ± 9.09 vs. 24.55 ± 12.28 (Δ% +36.64; *p* < 0.001; ES 0.75). Hamstring sub-analysis: higher TD (Δ% +19.28; *p* = 0.026), HSR (Δ% +25.38; *p* = 0.014), SR distance (Δ% +41.78; *p* = 0.002), sprint count (Δ% +34.69; *p* = 0.005), LACC (Δ% +18.36; *p* = 0.047), HDEC (Δ% +23.32; *p* = 0.031).	Muscle-tendon injuries (including hamstring strains) were preceded by a 1-week spike in accumulated external load across multiple running- and acceleration-based metrics, relative to each player's own prior 6-week “norm.”
Soler et al. (68)	Paired-sample *t*-tests (injury week vs. control). Significant increases in injury week: training volume (Δ% +25.11; *p* < 0.01; ES 3.18); TD (Δ% +19.63; *p* < 0.01; ES 2.04); LACC (Δ% +11.43; *p* < 0.01; ES 0.66); LDEC (Δ% +19.73; *p* < 0.01; ES 1.13); HACC (Δ% +18.60; *p* < 0.01; ES 0.84); HDEC (Δ% +26.58; *p* < 0.01; ES 0.96); HMLD (Δ% +30.72; *p* = 0.03; ES 0.60). HIR, SR, high SR, sprint count were not statistically different (*p* > 0.05).	Calf strain injuries were preceded by a week with unusually high training volume and total/accelerating/decelerating loads, suggesting a short-term overload pattern (particularly accel/decel-related exposure) in the week before injury.

NR, not reported; HSR, high-speed running; HSD, high-speed distance; TD, total distance; SR/SPD, sprint running/sprint distance; ACWR, acute:chronic workload ratio; EWMA, exponentially weighted moving average; AUC, area under the receiver operating characteristic curve; RR/OR/HR/IRR, risk/odds/hazard/incidence-rate ratio; CI, confidence interval; IMU, inertial measurement unit; GNSS/GPS, global navigation satellite system/global positioning system; HIR, high-intensity running; HMLD, high metabolic load distance; ACC/DEC, accelerations/decelerations.

## 4 Discussion

Across the evidence, the most consistent signal was not that more HSR is uniformly harmful, but that short-horizon increases or disproportionate exposure relative to recent history, particularly when chronic exposure is low, are more often reported to align with elevated injury risk than absolute weekly HSR volume *per se*. This pattern directly addresses the review aim of synthesizing associations and explaining heterogeneity, because it recurs across multiple exposure windowing approaches (acute vs. chronic contrasts) while diverging when absolute volumes are analyzed in isolation. This tendency emerged in studies using acute:chronic workload ratio (ACWR) constructs and in within-player injury-week vs. control-week comparisons. However, given that most included studies were judged at high overall risk of bias, predominantly due to confounding control and statistical analysis/reporting domains, these patterns should be interpreted as hypothesis-generating rather than as stable risk thresholds. Some studies reported null or negligible associations for distance-based HSR measures within typical load ranges, highlighting substantive heterogeneity that appears partly methodological (e.g., definitions, windows, modeling), partly contextual (e.g., match vs. training exposure, competitive level), and partly outcome-specific (e.g., injury type, tissue).

### 4.1 Acute–chronic models and spikes as the dominant analytic paradigm

A large proportion of included studies operationalized HSR/sprinting exposure using ACWR or closely related acute–chronic contrasts. In general, these studies aligned on the proposition that risk increases when acute HSR-related demands meaningfully exceed recent chronic exposure, although the strength of association depended on the measure and context. For example, Bowen (57) reported elevated non-contact injury risk with moderate-high accumulated high-speed distance over 4 weeks (>20 km/h), and Bowen (58) showed substantially higher non-contact injury rates when very high ACWR occurred in the context of low chronic exposure—most particularly for decelerations (very high ACWR decelerations in a low-chronic condition). Malone (16) further reinforced this theme by showing large week-to-week changes in HSR/sprint distance and high ACWR values corresponded to higher lower-limb injury odds, while moderate weekly exposures were comparatively protective, suggesting a classic “preparedness vs. spike” interpretation rather than a simple dose–harm relationship.

More recent work extended these acute–chronic concepts to different speed bands and multiple-comparison controlled modeling. Fousekis et al. (59) reported strong associations between non-contact injury risk and ACWR for both moderate-speed running (15–20 km/h) and sprinting (>25 km/h), with the most discriminative models retained after false discovery rate adjustment. Kim and Choi (60), in a collegiate players, similarly found that higher ACWR quartiles for match-derived total distance, high-intensity distance, and sprint bouts were associated with greater non-contact soft-tissue injury odds. These findings support the view that relative change measures capture a component of risk that absolute weekly volume may miss, especially where abrupt increases occur.

At the same time, there were important exceptions that temper overgeneralization. Enright et al. (61) found that accumulated and ACWR-based HSR/sprinting measures did not differentiate injury tissue type or severity, and Suarez-Arrones et al. (62) observed that an ACWR “spike” threshold was frequently exceeded in uninjured players while injuries occurred without exceeding the spike criterion, albeit in a very small sample with few injuries. These discrepancies point to two critical considerations, namely threshold-based spike rules may be insufficiently specific, and statistical power and event rates can materially influence observed associations and their stability.

Interpretation of ACWR findings requires caution because ratio metrics can induce artefactual associations via mathematical coupling (shared numerator/denominator structure) and spurious correlation, particularly when acute and chronic components are highly correlated by construction (63). ACWR estimates are also sensitive to analyst degrees of freedom, including window-length choice (e.g., 7:28 vs. alternatives), smoothing method (rolling average vs. EWMA), and the use of categorization into spike bins, each of which can change apparent effect sizes and inflate false-positive findings under multiple testing. Consequently, ACWR should not be interpreted as a causal parameter, where acute–chronic dynamics are of interest, regression-based approaches using separate acute and chronic terms (and their interaction) are often preferable to ratios because they avoid coupling while enabling non-linearity and effect-modification modelling (64).

### 4.2 Absolute weekly exposure: evidence for both risk and adaptation

Some studies analyzed absolute weekly HSR/sprinting exposure (or binary “high vs. low week” classification) rather than ratio constructs. Here, the evidence was mixed and, in some cases, suggestive of adaptation effects. Nobari et al. (65) reported higher injury odds and risk during high-load weeks across multiple external-load variables including sprint-related measures, consistent with a risk-from-high-exposure interpretation. In contrast, Bacon and Mauger (66) did not find higher overuse injury incidence with greater weekly HSR, with injury rates numerically lower in the high HSR group, an observation compatible with either protective adaptation or selection/availability bias (i.e., fitter/more resilient players accumulating higher HSR).

Malone et al. (16) provides an important bridge between these perspectives by explicitly showing a non-linear (U-shaped) relationship, wherein moderate weekly HSR/sprint exposure was associated with lower injury odds than very low exposure, but rapid increases were harmful. This aligns with the conceptual model that chronic exposure builds tolerance, while abrupt changes exceed tissue capacity. Within-player designs focusing on injury weeks similarly showed that injury occurrence may cluster around weeks of higher-than-usual external load. Raya-González et al. (67) reported widespread increases across running and acceleration measures in the week preceding muscle-tendon injuries (including hamstrings), and Soler et al. (68) found calf injuries preceded by higher training volume and acceleration/deceleration loads even when HSR/sprint running distances were not significantly different. Possibly, contextualized increases relative to an individual's baseline may be more informative than absolute volume alone.

Notably, most evidence supporting these interpretations arises from observational models and descriptive comparisons with substantial potential for residual confounding (e.g., prior injury, match minutes, fitness, selection/availability).

### 4.3 Match vs. training context and the under-capture of match load

A recurring source of heterogeneity is whether exposures reflect training only, match only, or both. Match contexts likely concentrate the highest-intensity running and decisive injury mechanisms. Massard et al. (69) quantified this explicitly, reporting substantially lower odds of injury in training compared with matches, while concluding that (within typical ranges) HSR loads had negligible association with injury risk relative to contextual factors. Lu et al. (70) emphasized an important methodological limitation, since although most injuries occurred during matches, external match load was not captured, constraining inference about the most etiologically relevant exposures. Guitart et al. (71) similarly observed the highest injury incidence on match day and elevated incidence in the days leading into matches, supporting the idea that microcycle structure and match demands are central to injury occurrence but often incompletely represented when analyses rely deeply on training loads. In contrast, studies that interrogated the immediate pre-injury match period offer some coherence, for instance Gregson et al. (72) found sprinting distance in the minute preceding injury was associated with higher odds of match muscle injury, and Aiello et al. (73) showed that hamstring injuries frequently occurred at high absolute speeds and high percentages of individual maximal speed, typically in phases of acceleration or deceleration around >25 km/h. These studies suggest that match-intensity exposures, and particularly the most intense bouts, may co-occur with or immediately precede injury events, while training and weekly measures may represent background readiness and cumulative stress. Accordingly, match-level (event-window) analyses primarily inform contextual and potentially proximal injury mechanisms and inciting circumstances, whereas training-load metrics more plausibly reflect longer-horizon exposure history, preparedness/adaptation, and cumulative stress that condition risk over time.

### 4.4 Which high-speed constructs appear most informative?

Across studies, the most informative constructs varied, but several themes emerged. First, sprinting or very high-speed exposure often appeared in associations with injury occurrence (59, 67, 72, 73) and, in at least one short-horizon analysis, injured players showed higher sprint distance per minute in the week before hamstring injury (74). Second, acceleration/deceleration-related measures may be particularly relevant for certain injury types and contexts. For instance Bowen et al. (58) highlighted deceleration ACWR effects when chronic exposure was low, and Soler et al. (68) observed calf injuries preceded by increased acceleration/deceleration loads even without significant HSR/sprint distance differences. Third, there is evidence that moderate-speed running bands can also matter, not only maximal sprinting. For instance Fousekis et al. (59) identified ACWR in 15–20 km/h distance as highly discriminative, suggesting that risk may relate to broader high-intensity locomotor demands, not exclusively top-end speed.

However, the evidence also indicates that distance-based HSR measures alone may have limited sensitivity in some contexts. Importantly, these null and negligible associations are themselves informative, suggesting practical limits to using distance-only speed metrics as stand-alone surrogates for the multifactorial mechanical and contextual determinants of injury. Nilsson et al. (75) found no consistent group-level differences in short-term training load measures preceding hamstring strain injuries, and Enright et al. (61) found HSR-related workloads did not distinguish injury tissue type or severity. These null findings likely reflect, at least in part, the multifactorial nature of injury and the limitations of summarizing complex mechanical exposure using only speed-derived distances.

### 4.5 Absolute vs. individualized thresholds: limited direct evidence of added value

Only a small subset of included studies directly compared absolute and individualized HSR thresholding approaches within the same dataset. Massard et al. (69) found that using an individualized threshold (>vIFT) vs. an absolute threshold (≥5.5 m/s) did not materially alter the interpretation of the acute–chronic HSR–injury models. Within the central observed load ranges, predicted effects were close to baseline and uncertainty increased at the extremes. Aiello et al. (73) provided complementary descriptive evidence using relative intensity (% of maximal speed), showing that hamstring injuries frequently occurred at high fractions of individual maximum, which conceptually supports individualization when interpreting exposure severity. Overall, the current evidence does not establish clear superiority of individualized thresholds for predicting injury, but it does suggest they may improve physiological interpretability and between-player comparability, particularly when maximal speed capacity varies or changes over time.

As suggestions, when individualized HSR/sprint thresholds are used, maximal sprint speed (MSS) should be defined using a reproducible protocol (e.g., radar/timing gates or validated GNSS procedures) and reported with device type, sampling frequency, and the operational definition of peak speed (instantaneous vs. averaged split). Because maximal sprint speed can change over a season and may be suppressed during fatigue or return-to-play phases, studies should prespecify an maximal sprint speed updating rule (e.g., best-of-season, rolling best over the prior 4–8 weeks, or scheduled re-tests) and report how missing/invalid observations were handled.

### 4.6 Study limitations and future research

The most recurrent methodological limitation was insufficient control of confounding, which, under QUIPS decision rules, was the dominant driver of the predominantly high overall risk of bias. Many investigations relied on largely unadjusted or weakly adjusted analyses (or descriptive profiling) despite the strong likelihood of confounding by factors such as prior injury, match exposure/minutes, fitness, positional role, and time-varying contextual load. This was evident in studies that were primarily descriptive or compared injury weeks to prior averages without multivariable control (67, 68, 71), in workloads modeled independently with authors acknowledging important unmeasured factors (57, 58), and in analyses using simple between-group comparisons (16, 74). Even where adjustment was attempted, it was often limited to a small covariate set (59), leaving substantial residual confounding likely. In contrast, only a minority used designs/analyses with stronger protection against between-player confounding [e.g., within-player conditional modeling (72)], though time-varying confounding still remained plausible.

Another common issue was selection/attrition and missing-data handling that plausibly produced informative missingness and exposure misclassification. Several studies excluded large proportions of injuries or datasets due to unavailable monitoring or inconsistent data (61, 75), which can distort exposure–injury associations if missingness relates to training participation, monitoring coverage, or injury severity. Others estimated/imputed missing external-load data (57, 58, 69, 75), sometimes transparently and with quality-control thresholds (69), but nonetheless introducing risk of misclassification if data were not missing at random. Exposure measurement limitations also arose when external load did not capture important contextual factors, most notably the absence of match GPS (70), a structural limitation that directly threatens the validity of HSR exposure measurement for prognostic inference. Moreover, outcome definitions and analytic units were sometimes misaligned with prognostic aims. For instance a study (60) used a broad self-reported “musculoskeletal pain/discomfort” outcome, while other (65) analyzed weekly team-level injury counts rather than player-level outcomes aligned to individual exposure, and several studies were underpowered (76, 77), increasing the likelihood of unstable estimates and optimistic inference. Finally, generalizability was frequently limited because most cohorts were drawn from single teams/clubs (67, 68, 70), constraining external validity and raising the possibility that observed associations reflect club-specific monitoring practices, training philosophies, or medical surveillance intensity. These limitations (confounding, missingness/selection, exposure misclassification, outcome/analytic misalignment, and limited external validity) directly motivate the cautious, non-threshold-based interpretation of the observed patterns reported in this review and the emphasis on hypothesis-generating rather than prescriptive conclusions.

Future studies should prioritize standardize and report exposure definitions (HSR/sprint thresholds, processing/filtering, windowing) and capture match load in addition to training, given that matches represent a high-risk context and are often under-measured in prognostic datasets and use models that explicitly address clustering and dependence (player, team, season; repeated measures; recurrent injuries), and report how within-player correlation was handled (e.g., mixed models, frailty/clustered survival). Moreover, future studies must address time-varying confounding (e.g., match minutes, fitness status, prior injury, selection/availability) using appropriate longitudinal methods.

### 4.7 Practical implications

Across the included studies, the most defensible practical implication is that monitoring systems should focus less on absolute HSR “dose limits” and more on detecting disproportionate short-term increases relative to established chronic exposure, particularly for sprinting and high-intensity running bands, and for acceleration/deceleration loads where relevant. The combined evidence supports a programming emphasis on progressive chronic exposure development—consistent with the protective signal of moderate habitual HSR/sprint loads (16)—while minimizing abrupt spikes (58–60, 67). At the same time, studies showing null effects within typical ranges (61, 69, 75) caution that HSR measures alone should not be treated as deterministic. Rather, they should be integrated with contextual information (match congestion, microcycle design, fitness status) and, where feasible, complementary indicators of tissue capacity and mechanical strain. Thus, implications should be interpreted as guiding principles for monitoring and programming rather than as universal thresholds, because current evidence does not support transportable cut-points across teams, devices, and player populations. Given that most included studies were at high overall risk of bias, justified predominantly by confounding control and statistical analysis/reporting domains, any apparent exposure–injury patterns should be interpreted cautiously as hypothesis-generating rather than threshold-defining evidence.

## 5 Conclusions

Low-certainty evidence suggests that the relationship between HSR exposure and injury outcomes in soccer is contextual and often non-linear, and confidence in any single estimate is limited by the predominantly high risk-of-bias profile of the included studies. Consistent with the review objective of synthesizing associations and explaining heterogeneity, the most reproducible pattern across the literature was that short-horizon increases or disproportionate exposure relative to recent history, particularly when chronic exposure is low, more often aligned with higher injury risk (prognostic modeling) and higher injury occurrence in event/context designs, whereas associations with absolute weekly HSR volume were more frequently null, inconsistent, or non-monotonic within typical ranges. Persistent heterogeneity across studies appears driven not only by divergent findings but also by recurrent methodological variation and limitations, including inconsistent injury and exposure definitions, incomplete capture of match load, heterogeneous operationalization of HSR/sprint metrics, and variability in modeling and confounding control. Accordingly, these associations should be interpreted as context-dependent and hypothesis-generating, rather than predictive, causal, or suitable for defining transportable HSR thresholds for injury prevention across teams, devices, and player populations.

## Data Availability

The original contributions presented in the study are included in the article/supplementary material, further inquiries can be directed to the corresponding author.
